# Compliance and Satisfaction for 16 months of Adapted Tango vs. Supervised Walking for People with Parkinson’s

**DOI:** 10.1038/s41531-025-01220-8

**Published:** 2025-12-26

**Authors:** Haneul Kim, Forouzan Rafie, Amir H. Nekouei, Meghan E. Kazanski, Madeleine E. Hackney

**Affiliations:** 1 Rollins School of Public Health, Emory University, Atlanta, GA USA; 2 Department of Medicine, Division of Geriatrics and Gerontology, Emory University, Atlanta, GA USA; 3 Modeling in Health Research Center, Institute for Futures Studies in Health, Kerman University of Medical Sciences, Kerman, Iran; 4 Department of Rehabilitation Medicine, Emory University School of Medicine, Atlanta, GA USA; 5 Atlanta VA Center for Visual & Neurocognitive Rehabilitation, Atlanta, GA USA; 6 Birmingham/Atlanta VA Geriatric Research Education and Clinical Center, Atlanta, GA USA

**Keywords:** Neuroscience, Psychology, Diseases, Psychology

## Abstract

The present study is an exploratory secondary analysis examining associations between Parkinson’s disease (PD) characteristics and compliance and satisfaction with exercise programs as part of ongoing clinical trial research. 36 participants with PD engaged in either adapted tango (AT; *n* = 20) or supervised walking (WALK; *n* = 16) classes for 16 months. This trial was registered at ClinicalTrials.gov (NCT04122690) on October 10, 2019. PD-related metrics, dyskinesia frequency and duration, OFF-time, freezing of gait (FOG), disease duration, Hoehn-Yahr stage, and motor and cognitive function were collected. Linear regression models assessed associations with attendance and satisfaction. Attendance varied widely (range: 1–76; mean ± SD: 39.1 ± 26.0 sessions), with overall satisfaction favorable (4.0 ± 0.8 on a 5-point scale). Dyskinesia metrics showed negative correlations with compliance: percentage of dyskinesia (*β* = –0.381, *R*^2^ = 0.145, *p* = 0.055) and total dyskinesia duration (*β* = –0.377, *R*^2^ = 0.142, *p* = 0.058). Compliance positively predicted satisfaction (*β* = 0.378, *R*^2^ = 0.143, *p* = 0.063). Montreal Cognitive Assessment (MoCA) was the strongest satisfaction predictor (*β* = 0.396, *R*^2^ = 0.157, *p* = 0.050), followed by the Movement Disorder Society-sponsored revision of the Unified Parkinson’s Disease Rating Scale (MDS-UPDRS) scores (*β* = –0.343, *R*^2^ = 0.118, *p* = 0.093). FOG had no significant effect on attendance or satisfaction. Findings indicate dyskinesia limits compliance, while cognitive function enhances satisfaction, emphasizing the need for tailored exercise.

## Introduction

Parkinson’s disease (PD) is a long-term neurodegenerative disorder. PD profoundly hampers both motor and cognitive abilities, greatly diminishing quality of life (QOL) and general well-being^1,2^. PD ranks as the second most prevalent neurodegenerative condition worldwide, impacting around 2% of individuals over the age of 65 years. The occurrence of PD is steadily rising due to the aging global population^3^. PD is caused by the degeneration of dopaminergic neurons in the basal ganglia and is characterized by the cardinal signs: tremor, bradykinesia, muscle rigidity, and postural instability. PD also has non-motor symptoms (NMS), such as depression, dysphagia, and urinary dysfunction^4^.

The manifestation of OFF episodes, defined as periods when the effects of medication wear off and motor symptoms worsen, greatly diminishes the individual’s QOL. The onset of OFF episodes represents a significant hurdle in the therapeutic management of PD^5^. Moreover, dyskinesias are involuntary motions triggered by prolonged use of levodopa. Both OFF episodes and dyskinesias lead to unpredictable fluctuations throughout the day, greatly hindering individuals’ ability to carry out everyday tasks and adversely impacting levels of physical activity^6^. Medications such as Levodopa can temporarily relieve symptoms, especially in the early stages of PD. The treatment plan can result in drug-resistant complications and the return of symptoms of motor cognitive decline as the condition progresses. Therapeutic strategies must be broadened to incorporate supportive interventions and lifestyle adjustments^7^.

Regular physical activity has emerged as a promising supportive approach to address PD symptoms and potentially slow the progression of the disorder^7^. Moderate aerobic exercise could improve motor skills, movement, and balance stability^7–9^. However, observational evidence suggests that greater levels of regular physical activity may be associated with a decline in motor and cognitive abilities^10^. Also, many current studies are constrained by brief intervention periods, typically lasting under six months, and a lack of extended follow-up assessments^11^.

Exercise has increasingly been recognized as an indispensable component of thorough PD treatment. Numerous investigations have demonstrated that routine physical activity alleviates both motor-related and non-motor-related issues. Routine physical activity also enhances overall QOL and could potentially slow the progression of the condition^12^. Despite the expanding body of evidence emphasizing these advantages, including participation in intervention programs, remains insufficiently addressed. This lack of uptake has led researchers to examine the fundamental motivational and structural aspects of influencing involvement.

Movement-related challenges, such as dyskinesia, Freezing of Gait (FOG), and OFF-time, can pose significant barriers to maintaining consistent physical activity. As reduced treatment efficacy during these intervals often worsens mobility impairments and diminishes motivation^13,14^. Additionally, logistical obstacles obstruct participation in exercise programs. These obstacles include transportation challenges, time limitations, and restricted access to exercise facilities, which also reduce the perceived value of fitness-focused programs^12^.

In a comprehensive review, Schootemeijer et al. (2020) identified four categories of enablers and obstacles to exercise engagement. These categories are followed by the framework of the International Classification of Functioning, Disability, and Health (ICF). The categories included bodily functions, tasks and involvement, individual traits, and environmental influences. Among the most notable physiological difficulties were exhaustion, muscle rigidity, involuntary shaking, indifference, and unease^12^. Ellis et al. (2013) conducted a study with 260 individuals diagnosed with PD. The research study determined three key deterrents to participating in physical activity. The list comprised low perceived advantages of exercise (OR = 3.93), insufficient time (OR = 3.36), and fear of losing balance (OR = 2.35), which were markedly more common among those who refrained from engaging in physical movement^15^.

Similarly, Hunter et al. (2019) conducted a qualitative systematic analysis. The researchers discovered that numerous individuals with PD worried that physical activity might exacerbate their symptoms or cause harm. However, such misconceptions were frequently addressed through targeted education. Supporting group settings also played a role since they emphasized the value of structured and safe exercise routines^16^.

This study considered the substantial evidence demonstrating the beneficial impact of physical activity on improving motor abilities and physical performance in individuals with PD. We recognized that OFF periods frequently disrupt daily routines and hinder participation in structured exercise programs^13,14^. As such, this research explores how condition-specific factors, particularly dyskinesia and OFF episodes, impact compliance with and satisfaction with exercise intervention programs for individuals with PD. Throughout a 16-month intervention, participants engaged in either a partner-supported, adapted tango (AT) or a structured and supervised walking regimen (WALK). We hypothesized that dyskinesia and severe OFF episodes would increase the disease burden on patients, possibly through increased stigma leading to withdrawal from social activities or decreased capacity for gainful participation in exercise. We therefore predicted that a greater burden of dyskinesias and increased duration or severity of OFF episodes would result in reduced compliance and satisfaction with exercise intervention programs. The findings from this investigation may contribute to the development of more inclusive, personalized recovery strategies that efficiently tackle both condition-specific restrictions and practical challenges. The results could improve PD patients’ well-being and overall life quality within this expanding medical community.

## Results

Descriptive demographic and clinical characteristics are summarized in Table 1. There were no statistically significant differences between AT and WALK groups in age, sex, race, disease duration, Hoehn and Yahr stage, or PD-related motor complications, including OFF-time and dyskinesia at baseline (*P* > 0.05). Compliance and satisfaction scores were also comparable at baseline. These findings confirm that randomization produced equivalent groups, allowing for an unbiased evaluation of intervention effects.

**Table. 1:** Demographic and Parkinson’s disease characteristics of participants in the WALK and AT groups

	AT (*N* = 20)	WALK (*N* = 16)	*p*
Age (y)	Mean (SD): 70.3 (7.7) Range: 49.0–83.0	Mean (SD): 71.1 (7.7) Range: 57.0–82.0	0.78
Sex (F/M)	Female: 6 (30%) Male: 14 (70%)	Female: 3 (19%) Male: 13 (81%)	0.44
Race	Asian: 1 (5%) Black/African American: 5 (25%) Hispanic or Latino: 1 (5%) Other: 1 (5%) White/Caucasian: 12 (60%)	Asian: 0 (0%) Black/African American: 4 (25%) Multicultural: 1 (6%) White/Caucasian: 11 (69%)	0.58
OFF (%)^◊^	Mean (SD): 12.4 (15.2) Range: 0.0–42.2	Mean (SD): 15.4 (17.6) Range: 0.0–46.5	0.69
Off duration^◊^	Mean (SD): 118.0 (143.7) Range: 0.0–380.0	Mean (SD): 150.0 (177.1) Range:0.0–470.0	0.66
Dyskinesia (%) ^◊^	Mean (SD): 4.4 (5.9) Range: 0.0–18.7	Mean (SD): 5.5 (8.9) Range: 0.0–27.0	0.75
Dyskin. duration^◊^	Mean (SD): 42.0 (61.2) Range: 0.0–200.0	Mean (SD): 51.0 (80.2) Range: 0.0–240.0	0.78
Hoehn-Yahr Stage	Mean (SD): 2.3 (0.5) Range: 1.5-3.0	Mean (SD): 2.1 (0.7) Range: 1.0–3.0	0.35
Years w/PD (y)	Mean (SD): 6.4 (4.8) Range: 0.0–15.0	Mean (SD): 8.5 (5.2) Range: 2.0–17.0	0.32
Compliance	Mean (SD): 61.1 (19.9) Range:21.0–85.0	Mean (SD): 57.0 (19.9) Range: 12.0–81.0	0.61
Satisfaction	Mean (SD): 4.0 (0.9) Range: 1.6–5.0	Mean (SD): 4.1 (0.6) Range: 3.0–5.0	0.85

Values are presented as mean (SD) or frequency (percentage). No statistically significant differences were observed between groups at baseline (all *p* > 0.05). ◊N-Miss: 3 for AT and N-Miss: 2 for WALK.

### PD-related clinical variables and program compliance

**Fig. 1:**
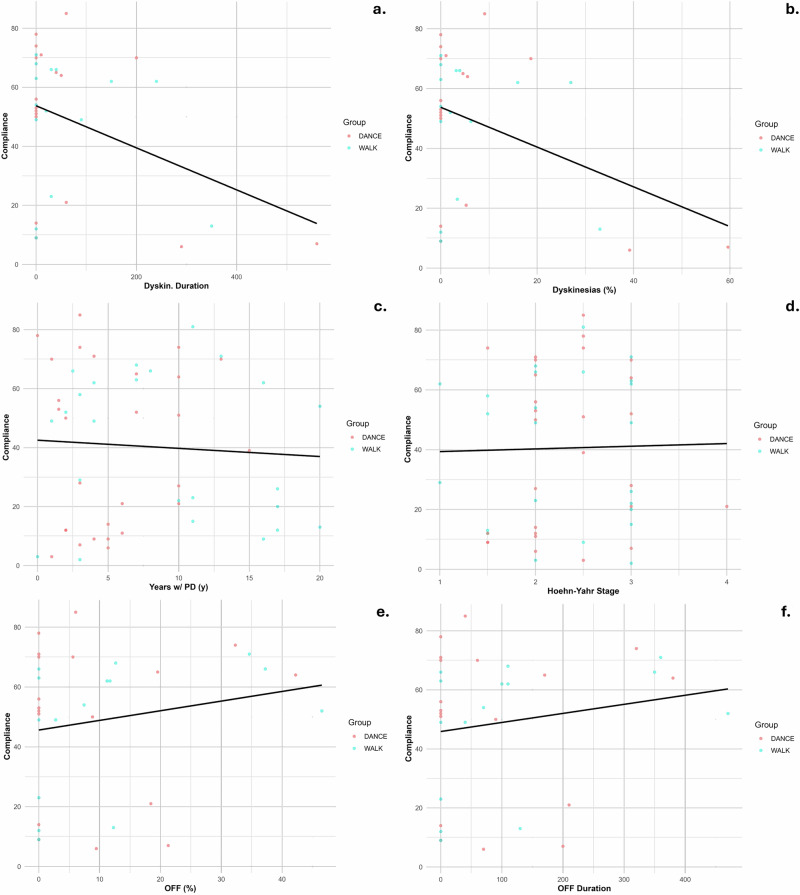
Relationship between Parkinson's disease characteristics and program compliance. Scatter plots show individual participant data with linear regression lines. **a** Dyskinesia duration vs compliance (*R*² = 0.142, *p* = 0.058); **b** Dyskinesia percentage vs compliance (*R*² = 0.145, *p* = 0.055); **c** Disease duration vs compliance (*R*² = 0.015, *p* = 0.473); **d** Hoehn–Yahr stage vs compliance (*R*² = 0.004, *p* = 0.731); **e** OFF-time percentage vs compliance (*R*² = 0.043, *p* = 0.312); **f** OFF-time duration vs compliance (*R*² =0.037, *p* = 0.347).

Dyskinesia percentage (*R*^2^ = 0.145, slope = −0.695, *p* = 0.055) and dyskinesia duration (*R*^2^ = 0.142, slope = −0.075, *p* = 0.058) exhibited the strongest negative trends with compliance. These trends suggest that individuals experiencing more severe or prolonged dyskinesias were less likely to be compliant with the program. The linear regression lines superimposed on the scatter plots underscore a strong negative relationship between dyskinesia severity and compliance (Fig. 1).

Conversely, other clinical variables demonstrated notably weaker associations. Duration of disease (*R*^2^ = 0.015, slope = −0.656, *p* = 0.473) and Hoehn-Yahr stage (*R*^2^ = 0.004, slope = 2.631, *p* = 0.731) showed negligible relationships with compliance. Similarly, both OFF-time percentage (*R*^2^ = 0.043, slope = 0.382, *p* = 0.312) and OFF-time duration (*R*^2^ = 0.037, slope = 0.036, *p* = 0.347) were minimally predictive of compliance (Fig. 1).

### PD-related clinical variables and program satisfaction

**Fig. 2:**
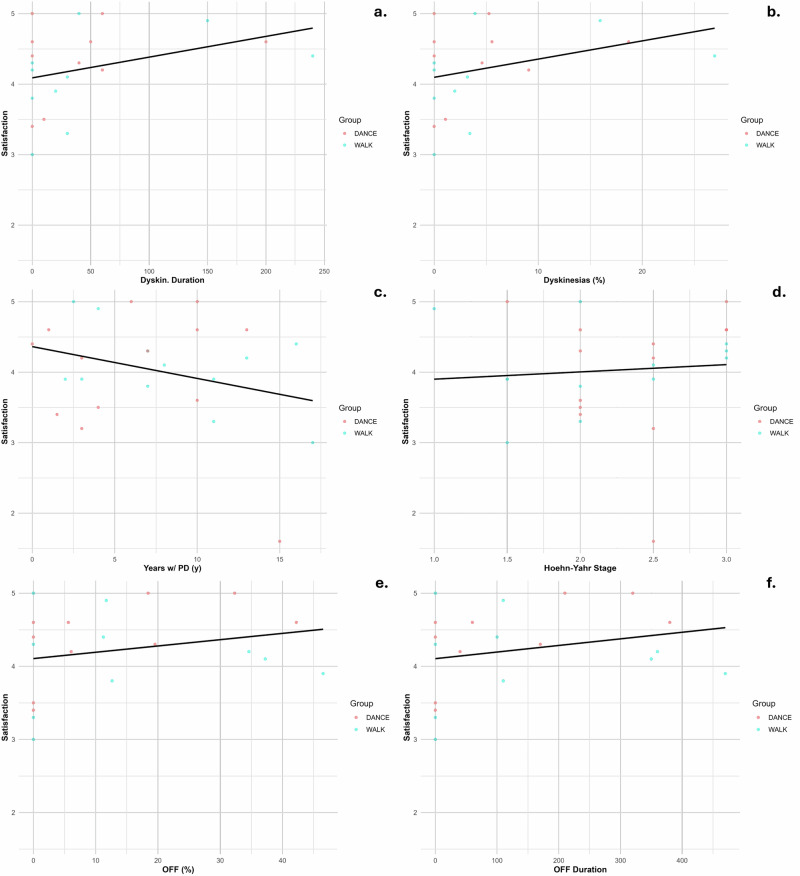
Relationship between Parkinson's disease characteristics and program satisfaction. **a** Dyskinesia duration vs satisfaction (*R*² = 0.120, *p* = 0.134); **b** Dyskinesia percentage vs satisfaction; **c** Disease duration vs satisfaction (*R*² = 0.086, *p* =0.155); **d** Hoehn–Yahr stage vs satisfaction (*R*² = 0.006, *p* = 0.712); **e** OFF-time percentage vs satisfaction (*R*² = 0.055, *p* = 0.318); **f** OFF-time duration vs satisfaction (*R*² = 0.058, *p* = 0.305).

Dyskinesia duration (*R*^2^ = 0.120, slope = 0.003, *p* = 0.134) exhibited the strongest positive trend with satisfaction. This trend suggests that individuals who experience more severe or prolonged dyskinesias are more likely to report higher program satisfaction. The linear regression lines superimposed on the scatter plots underscore a weak positive relationship between dyskinesia duration and satisfaction. Conversely, other clinical variables demonstrated notably weaker associations. Duration of disease (*R*^2^ = 0.086, slope = −0.045, *p* = 0.155) and Hoehn-Yahr stage (*R*^2^ = 0.006, slope = 0.103, *p* = 0.712) showed negligible relationships with satisfaction. Similarly, both OFF-time percentage (*R*^2^ = 0.055, slope = 0.009, *p* = 0.318) and OFF-time duration (*R*^2^ = 0.058, slope = 0.001, *p* = 0.305) were minimally predictive of satisfaction (Fig. 2).

### Association between satisfaction and compliance with motor and cognitive performance

**Fig. 3:**
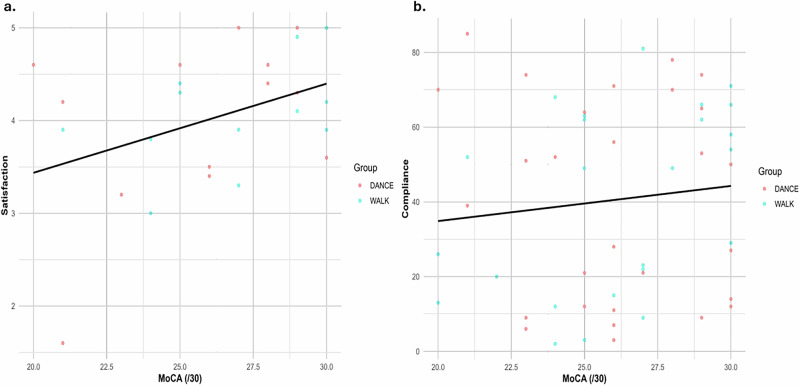
The relationship between satisfaction and compliance with MoCA. **a** MoCA vs satisfaction (*R*^2^ = 0.157, slope = 0.096, *p* = 0.050); **b** MoCA vs Compliance (*R*^2^ = 0.008, slope = 0.840, *p* = 0.601).

Linear regression analyses were conducted to examine the relationship between cognitive and motor performance indicators and program outcomes. The Montreal Cognitive Assessment (MoCA), a measure of global cognition, was a significant predictor of perceived program satisfaction (*R*^2^ = 0.157, slope = 0.096, *p* = 0.050) (Fig. 3a). This indicates that a participant’s ability to cognitively process and appreciate a program’s benefits could be a crucial factor in their overall positive experience. Conversely, MoCA scores had a negligible relationship with program compliance (*R*^2^ = 0.008, slope = 0.840, *p* = 0.601) (Fig. 3b).

**Fig. 4:**
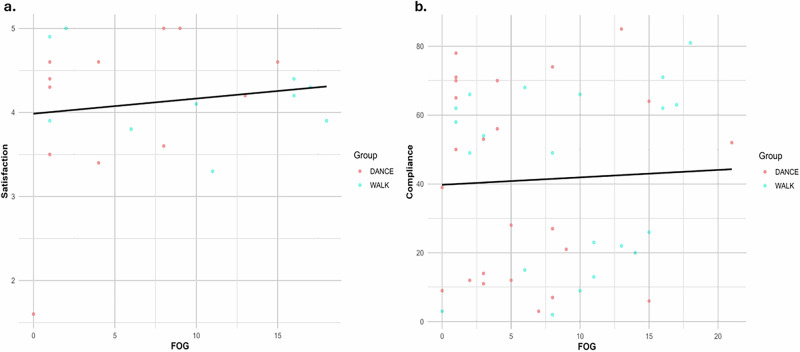
The relationship between satisfaction and compliance with FOG. **a** FOG vs satisfaction (*R*^2^ = 0.022, slope = 0.018, *p* = 0.514); **b** FOG vs compliance (*R*^2^ = 0.007, slope = 0.393, *p* = 0.665). Higher dyskinesia duration showed a weak positive trend with satisfaction.

In contrast, FOG showed no significant relationship with either satisfaction (*R*^2^ = 0.022, slope = 0.018, *p* = 0.514) (Fig. 4a) nor compliance (*R*^2^ = 0.007, slope = 0.393, *p* = 0.665) (Fig. 4b). These findings suggest that FOG was not a meaningful predictor for either outcome. Overall, the trends indicate that program satisfaction is more closely tied to a participant’s cognitive state, while compliance is likely influenced by other factors.

### Association between participant satisfaction and compliance

**Fig. 5:**
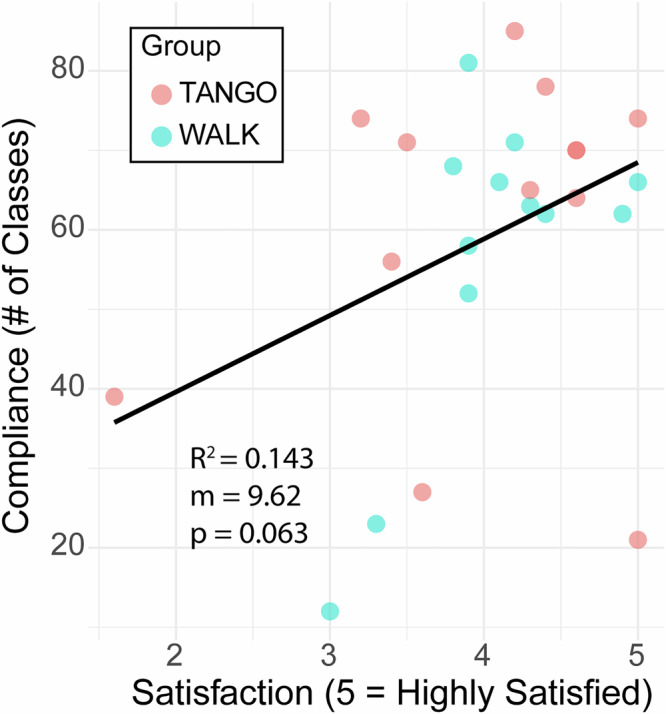
Association between program satisfaction and compliance. Scatter plot demonstrates a positive linear relationship (*R*² = 0.143, slope = 9.62, *p* = 0.063) between participant satisfaction ratings (*x* axis, scale 1–5) and total number of sessions attended (*y* axis). While the *p* value approaches significance, the trend suggests participants with higher satisfaction maintained better attendance throughout the 16-month program.

A positive linear relationship is apparent in Fig. 5, indicating that increased satisfaction is typically correlated with better attendance (*R*^2^ = 0.143, slope = 9.62, *p* = 0.063). While the *p* value (0.063) is just above the conventional threshold for statistical significance, it represents a strong, meaningful trend, suggesting that participants who perceived the program as more beneficial were more engaged (Fig. 5).

### Participant satisfaction profiles across different compliance and dyskinesias subgroups

**Fig. 6:**
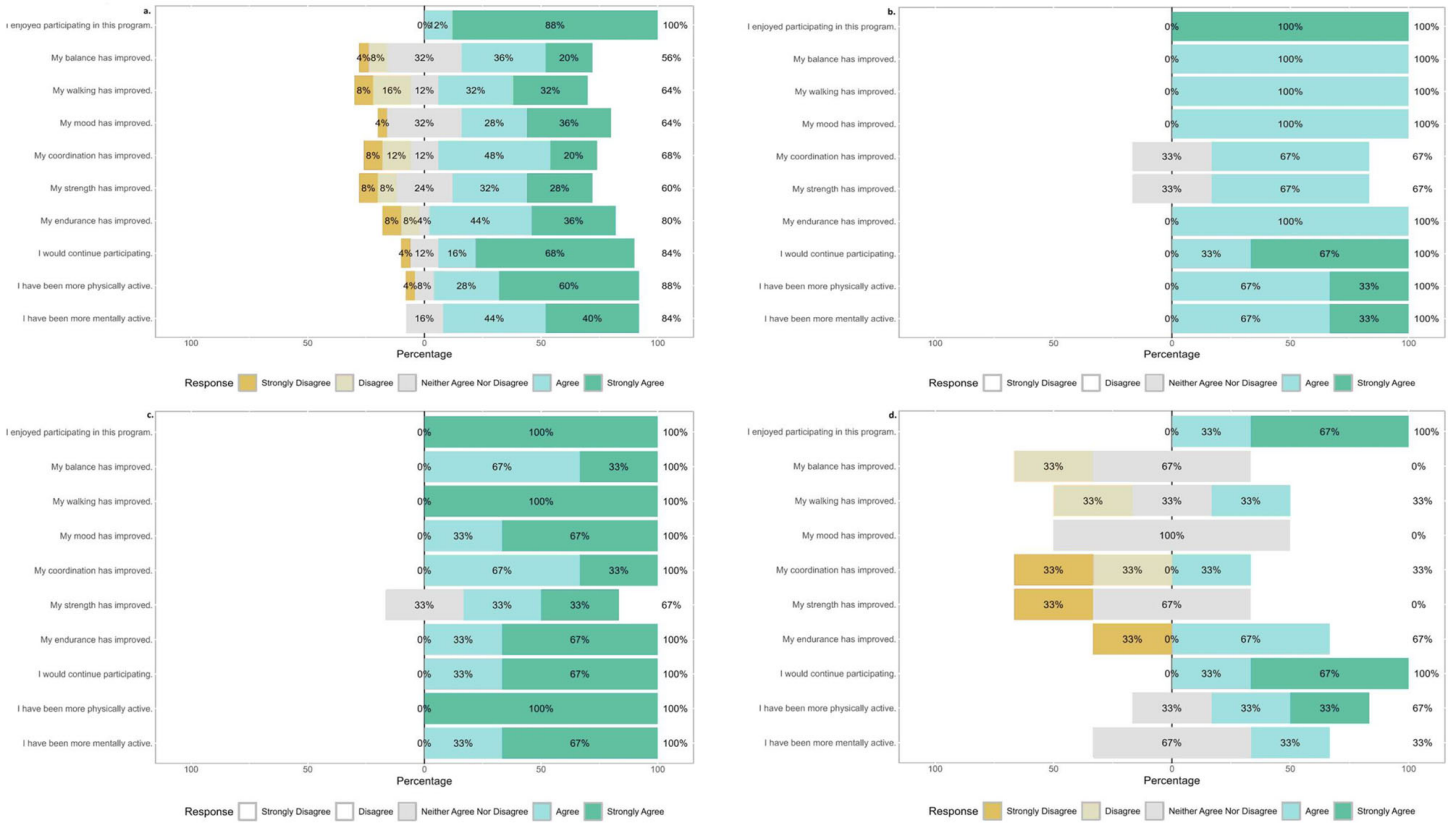
Participant satisfaction profiles across subgroups. **a** Overall satisfaction across all participants. **b** High compliance participants with low dyskinesia. **c** Moderate compliance participants with moderate dyskinesia. **d** Low compliance participants with low dyskinesia. Response distributions are shown as percentages for each questionnaire item across all panels.

In our study, all participants (100%) reported enjoying the program, and most (84%) indicated they would continue if given the opportunity. Participants expressed strong positive feedback regarding mental stimulation and mood improvement, with 84% affirming benefits in these aspects. However, perceptions of physical gains varied more widely. 64% agreed or strongly agreed they noted improved walking, whereas 24% disagreed or strongly disagreed that they noted improved walking (Fig. 6a). This finding highlights individual variability in perceived physical outcomes.

A subgroup of highly compliant participants with minimal motor complications reported consistently high satisfaction across all domains. All participants within this cluster endorsed enjoyment and willingness to continue the program. High ratings were also observed for improvements in mood, mental engagement, balance, and endurance. Figure 6b illustrates a generally favorable response pattern among highly compliant individuals with low dyskinesia (Fig. 6b).

Participant satisfaction data from participants characterized by both moderate dyskinesia and moderate compliance is illustrated in Fig. 6c. In this group, 100% of participants strongly agreed that the program contributed positively to various aspects of well-being, including walking ability, balance, mood, endurance, and overall physical and mental activity. While the majority (67%) strongly agreed that their strength had improved, 33% selected “neither agreed nor disagreed,” indicating slightly more variation in that perceived strength gains (Fig. 6c).

Participant satisfaction data from participants characterized by both low dyskinesia and low compliance is illustrated in Fig. 6d. While all participants in this group (100%) strongly agreed that they enjoyed the program, responses were more mixed regarding specific physical improvements. Only 67% of participants neither agreed nor disagreed that their balance had improved, with similar neutral or negative responses for walking, coordination, strength, and endurance (Fig. 6d). This suggests that enjoyment of the program may persist even in the absence of perceived physical benefit, particularly among less engaged participants.

## Discussion

The preliminary findings of this exploratory study examined the influence of PD-related clinical factors, including dyskinesia and OFF episodes, on participants’ engagement and satisfaction with two 16-month moderate-intensity cardiovascular programs: AT and WALK.

Our hypothesis and prediction that a greater burden of dyskinesias and increased duration or severity of OFF episodes would adversely impact compliance and satisfaction were supported by our findings. Our results showed that participants were highly satisfied with the activity program and reported positive experiences throughout its duration. Compliance was also strong, with the majority consistently attending sessions and following the prescribed exercises. Also, our findings highlighted the important role of PD-related characteristics, particularly motor and cognitive factors, in shaping both engagement and satisfaction. These disease-specific features appear to influence how participants experience and benefit from the program, underscoring the need to consider individual motor and cognitive profiles when evaluating compliance and satisfaction.

The study revealed that the severity of dyskinesia was a strong predictor of both program participation. On the other hand, the same PD-related factors as time since symptom onset, Hoehn and Yahr stage, and OFF status were found to be associated with participation and a weaker level of participation. Our initial hypothesis was confirmed: dyskinesias significantly limit physical activity participation. We found a negative correlation between dyskinesia severity and participation in the AT and WALK programs. This trend was the strongest association observed with compliance, approaching statistical significance for both dyskinesia percentage (*R*^2^ = 0.145, *p* = 0.055) and dyskinesia duration (*R*^2^ = 0.142, *p* = 0.058).

These results are consistent with a study by Hackney and Earhart that investigated the impact of motor symptoms, including dyskinesia, on exercise participation in people with PD^21^. Their study found that people with more severe motor symptoms, such as dyskinesia, had significant difficulties in adhering to exercise routines. Hackney and Earhart highlighted the need for tailored interventions, including flexible scheduling, tailored support, and alternative exercise-based therapies, to effectively engage people with PD who frequently experience long-term dyskinesia. From a mechanistic perspective, movement fluctuations, particularly dyskinesias, may interfere with participation by interfering with movement planning and increasing fall risk^5^. Our findings support the “dual-task interference” hypothesis, in which movement complications exacerbate cognitive-motor trade-offs, particularly in complex activities such as dancing^3^.

This observation aligns with previous research indicating that dyskinesia and functional mobility have a strong impact on daily physical activity engagement^28,29^. Participants with more pronounced dyskinesia showed lower attendance rates, whereas those with milder symptoms demonstrated a range of participation patterns, from regular involvement to occasional attendance. The variability in attendance emphasizes the subtle influence of symptom severity on compliance. These results reinforce prior research indicating that non-motor factors, particularly motivation, significantly influence compliance to exercise programs for individuals with PD^13^. Addressing these barriers is critical to improving participation rates, as low expectations of outcomes, fear of falling, and time constraints remain persistent challenges^13^.

In line with the findings of Politis et al. (2010), dyskinesia emerged as a significant obstacle to participation and was associated with reduced QOL and functional abilities^5^. These results provide additional evidence of reinforcing earlier studies, such as those by Politis et al. (2010), which associate dyskinesia with diminished QOL and reduced functional capabilities. The erratic nature of dyskinesia can prompt patients to experience involuntary movements during exercise, potentially disrupting their workout routines. Interestingly, our results are consistent with those of Mantri et al. (2021), who identified challenges in sustaining exercise routines, rather than lack of access, as the primary obstacle to participation. The extended duration of our intervention could account for the differences in outcomes observed between the studies^30^.

Although dyskinesia may be a barrier to participation, FOG does not significantly affect exercise participation. In addition, our results show that the severity of FOG cannot predict compliance or satisfaction with moderate aerobic exercise. Contrary to hypotheses, Hoehn and Yahr stage and disease duration did not significantly correlate with participation levels. Our results suggest that movement complications, particularly dyskinesias, may play a more important role than overall disease severity in predicting compliance.

While dyskinesia presents an intrinsic barrier, extrinsic motivators such as peer support and group camaraderie may help offset its negative emotional impact, fostering a more inclusive exercise environment. For instance, Schootemeijer et al. (2020) showed that group camaraderie enhanced compliance by alleviating the distress associated with motor fluctuations, indicating that social support can help cushion the emotional challenges of dyskinesia^12^.

The exit survey results indicated that participants in both intervention groups reported high levels of satisfaction and had overall positive experiences. The AT group, characterized by its interactive and social aspects, appeared to foster stronger mental engagement and emotional fulfillment, especially among individuals with moderate dyskinesia. The intricate nature of the dance, coupled with the social connections inherent in the activity, likely enhanced enjoyment and cognitive stimulation. This observation aligns with previous studies emphasizing the advantages of comprehensive exercise programs for people with PD^31^. In contrast, the WALK group offered a straightforward and predictable exercise alternative, appealing to those who preferred less strenuous activity. While walking ensured steady attendance, participants with milder dyskinesia displayed varied progress, particularly in areas like balance and stamina. These differences underscore the critical need for a variety of therapeutic options tailored to individual preferences and requirements, taking into account the motor function difficulties associated with PD.

Our findings here align with those of Hackney and Bennett^22^, who highlighted the significance of aerobic exercise programs for individuals with PD. Their work suggested that incorporating both social and physical components into therapeutic approaches enhances patient commitment and involvement. Moreover, McKay et al. (2016) identified that intricate activities like dance can boost cognitive engagement, potentially explaining the heightened satisfaction and mental stimulation reported by participants in our AT cohort^31^. The interactive aspect of AT, which required cooperation between partners, likely played a crucial role, delivering not only physical exercise but also opportunities for social connection. Social interaction alone may have positively impacted mood and overall participation, as evidenced by our findings. Studies have demonstrated that the social element of physical activity, particularly in group environments, is a significant driver of sustained involvement over the long term. Interaction with peers, the sense of community, and the formation of social bonds are essential for maintaining engagement in physical activities^32^. These insights underline the importance of weaving these components into exercise interventions for individuals with PD, promoting lasting commitment and participation.

Cognitive abilities (assessed via MoCA scores) showed the most notable factors associated with satisfaction. The findings suggest that, in addition to motor fluctuations, cognitive difficulties play a crucial role in shaping how participants perceive the advantage of the exercise interventions. However, MoCA scores were not significantly associated with compliance, indicating that cognitive capacity alone does not strongly influence compliance with the exercise program. Although dyskinesia severity consistently emerged as the strongest predictor of compliance and satisfaction, the influence of cognitive capacity and the burden of NMSs underscores the complex and multifaceted nature of exercise outcomes in PD. These insights point to the importance of designing exercise interventions that address motor challenges while also incorporating cognitive engagement and adaptable structures to meet the diverse symptoms and progression levels of participants.

This study investigated the association between satisfaction and compliance within both intervention groups. The results are consistent with previous research by Hauser et al. (2004), which found that satisfaction was closely related to the patient’s perceived intervention effectiveness throughout the day, making it an important measure for assessing outcomes^24^. The evaluation uncovered a favorable association between contentment and compliance, suggesting that higher satisfaction could be linked to increased participation. However, this relationship did not reach statistical significance, implying that other influences, such as external challenges or individual drive, may affect commitment. Furthermore, Hunter (2019) highlighted that negative experiences or observing patients with severe symptoms may lead to a reluctance to continue exercise^16^. This highlights the importance of supportive and social environments, consistent with previous research^33,34^. Our intervention, comprising the AT program and WALK, incorporated these principles by providing social interaction, instructor guidance, and an enjoyable atmosphere. These observations suggest that a positive exercise environment can enhance motivation and satisfaction.

Participants with high compliance and low dyskinesia reported comprehensive improvements across balance, gait, coordination, strength, stamina, moods, and overall mental engagement. These outcomes align with previous research conducted by Rafferty in 2017^35^, emphasizing the holistic benefits of structured physical activity in PD populations. Also, Carmo et al. (2024) have previously highlighted that exercise programs tailored to an individual’s symptoms, fitness level, and preferences are particularly effective for patients with PD, as they can enhance both participation and satisfaction^36^.

The elevated satisfaction levels highlight the program’s efficacy in delivering substantial motor and cognitive improvements for individuals with PD. Additionally, our data demonstrate that participants with greater compliance reported higher satisfaction, emphasizing the critical role of regular involvement in achieving better outcomes^17^. Aligning with previous research, our results indicate that individuals with less frequent dyskinesia were more likely to comply with the program, highlighting the necessity for personalized support to encourage participation among those experiencing more severe dyskinesia^9^.

Participants with moderate dyskinesia reported high levels of satisfaction, with all respondents strongly agreeing that they enjoyed the program. These positive feelings extended across a range of domains, including agreement with the statements that participants noted improved balance, walking ability, mood, endurance, and mental performance. However, opinions regarding improvements in coordination and strength were more mixed, with 67% strongly agreeing. These results are consistent with Cavanaugh (2015), who demonstrated that exercise programs for individuals with PD generally yield beneficial outcomes, although the degree of benefit may vary with disease severity^6^. For those managing moderate dyskinesia, this program seemed to provide meaningful support for both physical and mental well-being. These results highlight the importance of sustaining and expanding exercise programs for people with PD, while tailoring interventions to individual needs. Adjusting for varying motor functions and disease stages ensures that programs remain effective, engaging, and accessible for participants with diverse symptom profiles^6^.

Although all participants (100%) with low compliance and low dyskinesia unanimously agreed that they enjoyed the program, opinions on specific improvements were more divided. 67% strongly affirmed, and 33% expressed neutrality, that their level of physical activity had increased. This response suggests that limited participation may have reduced the perceived impact of the program, even in the absence of severe motor symptoms. The low attendance and diverse perspectives on program benefits highlight the critical need for individualized support strategies. Such approaches should aim to address specific challenges encountered by participants, tailoring the exercise program to accommodate each individual’s distinct motor and cognitive capabilities to enhance the program’s overall effectiveness.

Previous research has shown that non-clinical factors also play a substantial role in limiting participation^12^. Additionally, Ellis (2013) highlighted deterrents such as a “fear of falling,” “insufficient time,” and “low expectations regarding the effectiveness of exercise,” which are similar to our findings. These barriers underscore the importance of therapists addressing patients’ perceptions and convictions about the advantages of exercise when developing tailored interventions^15^.

This study identifies key factors influencing participation in exercise programs among individuals with PD. Notably, it highlights dyskinesia as a critical determinant of exercise compliance. In addition, our findings underscore the importance of developing more impactful and personalized exercise interventions that can enhance well-being and alleviate disease-related symptoms.

Because this was an exploratory study, the findings should be considered preliminary. Future studies with larger cohorts, including subgroups, such as patients with severe dyskinesia, may provide deeper insight into these complexities. The high reported level of participant satisfaction in the current study, while encouraging for the quality of the intervention, may be influenced by clinical and psychological factors, as well as other differences such as cultural, racial, ethnic, and socioeconomic.

While our study focused primarily on motor-related compliance and satisfaction measures, it is important to acknowledge that NMS such as sleep disorders, depression, anxiety, and cognitive impairment are prevalent in PD and may also influence exercise participation and perceived benefits. Future research should systematically evaluate how these often-overlooked symptoms affect compliance and satisfaction with structured exercise programs.

We evaluated symptoms of apathy using the Unified Parkinson's Disease Rating Scale (MDS-UPDRS) Part I. This method is supported by recent research from our group^37^, which found that apathy-related symptoms can emerge early in PD, with over 40% of individuals displaying apathy-related characteristics even in the mild stages of the condition. Given the high prevalence of both depression and apathy in PD, which can impact participation in exercise, future analyses can consider more specifically the impact of both apathy and depression on compliance and satisfaction.

Despite documented gender differences in the clinical manifestation of PD, the current study did not perform a specific analysis of differences in compliance or satisfaction between men and women. We acknowledge this omission. However, our prior work on a similar cohort from the southeastern United States, who have cultural, sociological, and racial/ethnic differences from other regions of the country^38^, found that men with PD experience significantly greater PD-related motor and non-motor burden compared to women. Given this established clinical context, we strongly recommend that future research explicitly investigate gender-based disparities as potential modulators of exercise compliance and satisfaction.

In the present study, caregivers were not directly enrolled in the intervention; thus, we lack outcome measures related to caregiver burden. We believe that this program could have benefits for caregivers. Our work shows that an AT program can benefit those who care for people with dementia, with promise in positively influencing aspects of the caregiving experience^39^. Future studies could specifically examine the effects of such exercise interventions on caregivers' well-being and caregiving burden.

These findings collectively highlight a complex relationship between PD medication-related motor fluctuations and freezing of gait and participant engagement in moderate-intensity aerobic programs. While participants reported high satisfaction with approaches like AT and WALK, program compliance varied significantly, primarily due to motor complications, especially dyskinesia. In contrast, cognitive function, as assessed by MoCA scores, showed a strong correlation with higher satisfaction levels. Importantly, FOG did not significantly impact program satisfaction or compliance, suggesting it may not hinder engagement in these moderate-intensity aerobic programs for PD. These findings underscore the critical need for tailored exercise programs that address both cognitive and motor symptoms to enhance compliance and maximize therapeutic outcomes for individuals with PD.

Future work should compare walking, dancing, and other evidence-based non-pharmacological interventions such as yoga, tai-chi, and music therapy to determine their relative effectiveness for motor and NMS in PD. Additionally, future research should investigate the potential effects of these interventions on common NMS, including sleep disorders, mood disorders, pain, and cognitive function, as these symptoms significantly impact QOL in individuals with PD.

## Methods

### Participants and recruitment

Participants were recruited through the Atlanta VAMC Movement Disorders Clinic using the VA Informatics and Computing Infrastructure (VINCI) database to identify eligible individuals within the Atlanta VA Health Care System. Additional participants were enrolled from the Emory University Movement Disorders Clinic, as well as through local PD support groups, educational events, and community outreach programs. At enrollment, participants underwent baseline assessments including general health screening, fall risk evaluation, demographic data (age, education), functional independence (activities of daily living), and global cognitive status using the MoCA. Participants were asked to continue their usual medical care and medications throughout the study.

### Inclusion and exclusion criteria

To be included, potential participants needed to present with a diagnosis of idiopathic PD, which was confirmed by a board-certified neurologist specializing in movement disorders, according to established diagnostic criteria^18^. To participate, they must be over 40 years of age and belong to Hoehn and Yahr stages I to III, which represent the severity of PD. The key requirement is an OFF period with a score of 1 or higher on section 4.3 of the MDS-UPDRS-IV. This score represents the period during which the drug’s effectiveness decreases^17^.

The diagnosis of PD was confirmed using the ICD-10 code “G20” and further supported by clinical signs, such as unilateral onset and the presence of at least three key symptoms: rigidity, bradykinesia, tremor, or postural instability. A documented favorable response to antiparkinsonian treatments was also an inclusion criterion that needed to be fulfilled to validate the diagnosis^19^. Individuals who received a Montreal Cognitive Assessment (MoCA) score at intake below 18, indicating moderate to severe cognitive dysfunction, were excluded^20^.

### Informed consent and participant flow

**Fig. 7:**
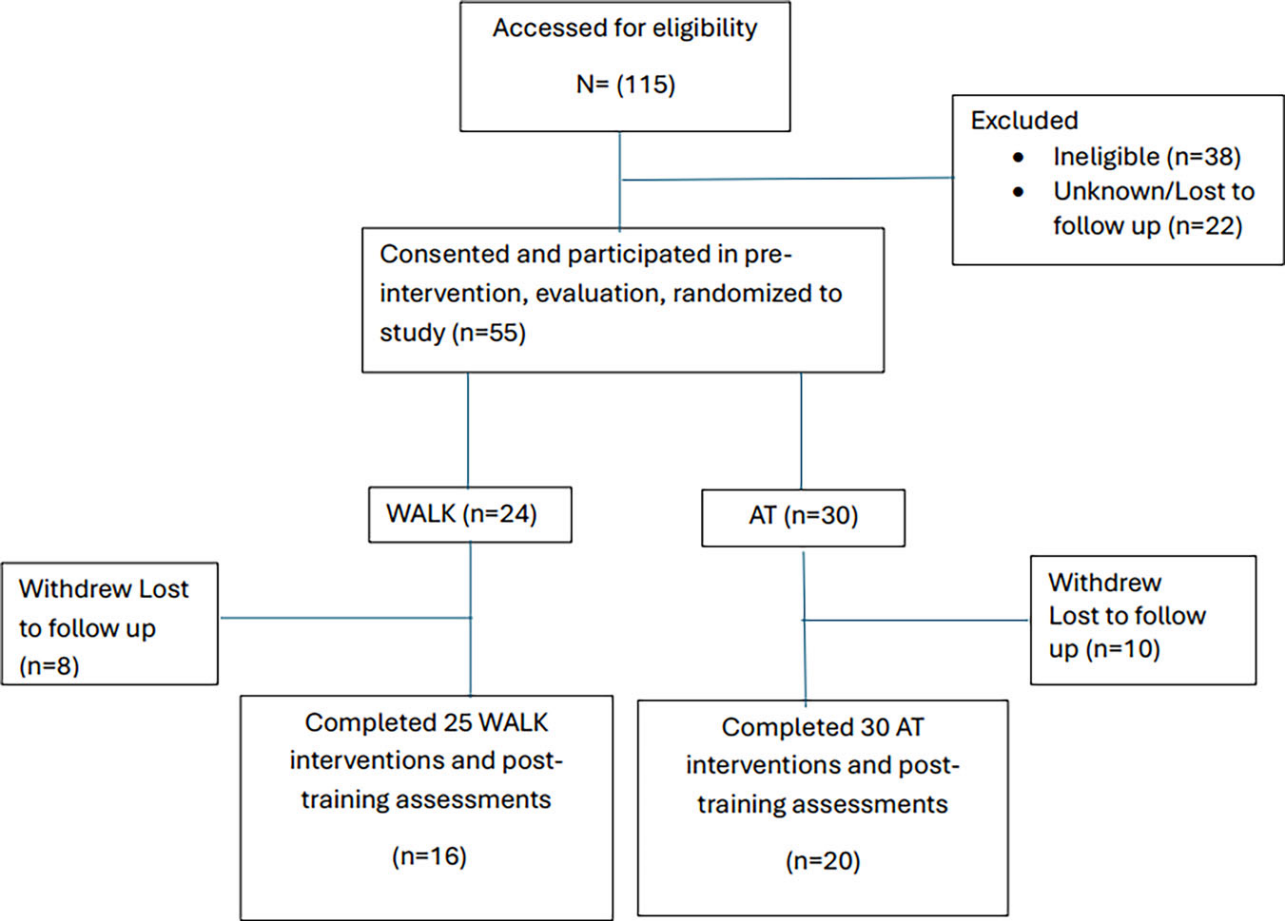
Consort diagram at the time of data analysis for this report. Flow diagram illustrating article allocation to the intervention adapted tango (AT) vs. supervised walking (WALK) over 16 months.

All participants provided written informed consent prior to study enrollment. Participant flow through the ongoing study is illustrated in Fig. 7 (CONSORT diagram).

### Randomization and intervention assignment

Thirty-six participants who met all inclusion and exclusion criteria were enrolled and randomly assigned to one of two intervention groups: the AT Group (*n* = 20), which participated in partnered dance-aerobic exercise sessions, or the WALK Group (*n* = 16), which participated in a supervised, structured walking program. Participants engaged in their assigned exercise interventions over 16 months. Both interventions were delivered over 16 months and consisted of two distinct phases. Participants attended bi-weekly sessions for the initial 3-month Training Phase, for a total of 24 lessons, each lasting 90 minutes, resulting in approximately 180 minutes of moderate aerobic activity per week. For the remaining 13 months, during a Maintenance Phase, participants came less often and engaged in weekly sessions. During the maintenance, the target of all participants was to participate in at least three sessions per month, amounting to 39 classes over the course of 13 months, with each meeting lasting 90 minutes. In total, the participants were to attend at least 63 sessions over the 16 months, but if they attended ~4 weekly sessions during Maintenance months, they would attend as many as 76 sessions.

The intervention was based on an adapted Argentine tango “AT” program that has been previously shown to improve motor function, balance, gait, mobility^21^, endurance, and QOL in individuals with PD^22^. Participants with PD were paired with non-PD partners such as trained caregivers, university students, or friends. Partners rotated every 15 minutes, and group sizes were capped at six pairs to prioritize safety and individualized attention. Sessions centered on exploring movement objectives through physical interaction, examining the connection between motion and rhythm, introducing fresh dance techniques, and integrating mastered and creative components. The instructional approach generally had minimal focus on memorization of fixed routines. Instead, each class introduced new steps to foster motor skill development and sustained engagement.

The supervised outdoor walking group received an equivalent duration, frequency, level of intensity, degree of oversight, and access to the same facility and trainers as the AT group. Each WALK session consisted of a 25-minute warm-up, 45 minutes of continuous walking (with breaks as needed “ad libitum” on a per participant basis), and a 10-minute cooldown, with brief, integrated pauses for transitions between phases. WALK treatment was most often conducted outside on level ground, with indoor hallways used occasionally during unfavorable weather conditions. Both interventions (AT and WALK) took place in a group format and were supervised by skilled researchers and volunteers to maintain safety and foster social engagement.

### Assessment and measures

Disease severity and motor fluctuations were evaluated using the MDS-UPDRS I–IV by an MDS-certified rater blinded to participant allocation^23^. The MDS-UPDRS is composed of four sections: Part I examines non-motor experiences of daily living, Part II concerns motor-related experiences in daily living, Part III evaluates motor performance through a rated physical assessment, and Part IV concentrates on medication-related motor complications such as dyskinesia and OFF episodes. In this study, item 3 of MDS-UPDRS Part IV (time spent in the OFF state) was used as the primary outcome measure to quantify the severity of OFF time. Each item is scored on a scale from 0 to 4, with higher values indicating greater disability. Scores were recorded at each evaluation point and were used to analyze the relationship between compliance and disease progression.

To complement clinician-rated assessments, a three-day OFF state diary^24^ was administered monthly. Participants were instructed to categorize their motor state (OFF, ON, ON with disruptive dyskinesia, or sleep) and record every 30 minutes for 3 consecutive days. To measure off-time, we determined the ratio of waking hours spent in the OFF and ON states accompanied by disruptive movement disorders. This self-reported approach reduces bias and accurately captures symptom fluctuations throughout the day. However, challenges such as compliance and memory limitations persist^25,26^. Integrating the MDS-UPDRS Part IV with the diary was intended to provide a comprehensive, peer-reviewed assessment of the intensity and complexity of OFF time.

Cognitive status was assessed using the MoCA, a widely validated screening tool for detecting mild cognitive impairment in individuals with PD. Motor symptoms, including dyskinesia and FOG, were assessed using the full MDS-UPDRS I–IV. Dyskinesia burden was evaluated both as a percentage of waking hours and total daily duration, informed by both clinician scoring and diary data. FOG severity was measured using the Freezing of Gait Questionnaire^27^, a six-item instrument scored on a 24-point scale, with higher scores reflecting more frequent or disabling episodes. The disease stage was classified via the Hoehn and Yahr scale, which categorizes PD severity from stage 1 (mild symptoms) to stage 5 (severe symptoms requiring a wheelchair or resulting in being bedridden). These reliable assessments allowed for the measurement of both motor and NMS load pertinent to engagement in the intervention.

Gait and falls risk were assessed through the Gait and Falls Questionnaire, a 16-item tool evaluated on a 64-point scale (each item assigned a score from 0 to 4), with higher values reflecting an increased likelihood of falls. This assessment provided further understanding of mobility challenges linked to the progression of the disease.

At the conclusion of the intervention phase, participants engaged with a satisfaction questionnaire devised to evaluate subjective effects of program compliance. This instrument explored multiple dimensions, including perceived advancements in motor function and cognitive improvements, emotional resilience, and psychosocial benefits. Responses were gathered via a 5-tier Likert scale (1 = strongly agree to 5 = strongly disagree). This instrument was developed to evaluate self-reported outcomes that extend beyond clinician-led assessments or performance-based metrics. The resulting data provided valuable insights into the program’s acceptability, feasibility, and perceived significance from the participants’ perspective.

### Statistical analysis

All analyses were conducted in RStudio 4.4.1. Descriptive statistics summarized baseline and outcome variables. Between-group comparisons for continuous variables were performed using independent *t* tests, and categorical variables were assessed via chi-square or Fisher’s exact tests, as appropriate.

Associations between clinical variables (e.g., OFF-time, dyskinesia) and outcomes (compliance, satisfaction) were analyzed using Pearson correlation coefficients. For exploratory analyses, we identified associations that approached statistical significance (e.g., proportion and duration of dyskinesia). In addition, we utilized linear mixed-effects models to assess change over time, incorporating fixed effects for time, group, and group × time interactions, and a random intercept to allow participants to account for within-person variability. We provided the coefficient of determination (*R*^2^) for each model to measure explanatory power. Statistical significance was defined as 0.05, and marginal results (e.g., *p* = 0.055) were recognized as having potential clinical significance. The data analyst who performed the statistical analyses was blinded to group allocation.

### Ethical approval and trial registration

This study was conducted as an exploratory investigation and was approved by the Institutional Review Board of Emory University and the Research and Development Committee of the Atlanta Veterans Affairs Medical Center (VAMC). The trial was registered at ClinicalTrials.gov (NCT04122690) on October 10, 2019, with full protocol details previously published^17^. As of the drafting of this report, this study has not been completed for all enrolled patients; therefore, this manuscript presents preliminary results from our investigation, and any interpretation of results should be made with caution.

## Supplementary information

Checklist

## Data Availability

The data supporting this study's findings are available upon request.
