# Supplementary material for: Compliance and Satisfaction for 16 months of Adapted Tango vs. Supervised Walking for People with Parkinson’s
Source: NPJ Parkinsons Dis. 2025 Dec 26;12:13. doi: 10.1038/s41531-025-01220-8 (PMC12796157; doi:10.1038/s41531-025-01220-8)
Supplement: Supplementary file 1 — Checklist [file 41531_2025_1220_MOESM1_ESM.pdf]

Section/topic	No	CONSORT 2025 checklist item description	Reported on page no.
**Title and abstract**			
Title and structured abstract	1a	Identification as a randomised trial	Pg 2, 12
	1b	Structured summary of the trial design, methods, results, and conclusions	Pg 2
**Open science**			
Trial registration	2	Name of trial registry, identifying number (with URL) and date of registration	Pg 2, 12
Protocol and statistical analysis plan	3	Where the trial protocol and statistical analysis plan can be accessed	Pg 14
Data sharing	4	Where and how the individual de-identified participant data (including data dictionary), statistical code and any other materials can be accessed	Pg 14
Funding and conflicts of interest	5a	Sources of funding and other support (eg, supply of drugs), and role of funders in the design, conduct, analysis and reporting of the trial	Pg15
	5b	Financial and other conflicts of interest of the manuscript authors	Pg 15
**Introduction**			
Background and rationale	6	Scientific background and rationale	Pg 3-4
Objectives	7	Specific objectives related to benefits and harms	Pg 13
**Methods**			
Patient and public involvement	8	Details of patient or public involvement in the design, conduct and reporting of the trial	No public involvement
Trial design	9	Description of trial design including type of trial (eg, parallel group, crossover), allocation ratio, and framework (eg, superiority, equivalence, non-inferiority, exploratory)	Pg12
Changes to trial protocol	10	Important changes to the trial after it commenced including any outcomes or analyses that were not prespecified, with reason	No changes
Trial setting	11	Settings (eg, community, hospital) and locations (eg, countries, sites) where the trial was conducted	Pg12
Eligibility criteria	12a	Eligibility criteria for participants	Pg 12
	12b	If applicable, eligibility criteria for sites and for individuals delivering the interventions (eg, surgeons, physiotherapists)	Pg12-13
Intervention and comparator	13	Intervention and comparator with sufficient details to allow replication. If relevant, where additional materials describing the intervention and comparator (eg, intervention manual) can be accessed	Pg12-13
Outcomes	14	Prespecified primary and secondary outcomes, including the specific measurement variable (eg, systolic blood pressure), analysis metric (eg, change from baseline, final value, time to event), method of aggregation (eg, median, proportion), and time point for each outcome	Pg14
Harms	15	How harms were defined and assessed (eg, systematically, non-systematically)	No harms
Sample size	16a	How sample size was determined, including all assumptions supporting the sample size calculation	Not applicable
	16b	Explanation of any interim analyses and stopping guidelines	No interim analyses and stopping guidelines
Randomisation:			
Sequence generation	17a	Who generated the random allocation sequence and the method used	Pg 12
	17b	Type of randomisation and details of any restriction (eg, stratification, blocking and block size)	Pg 12
			**Reported on page no.**

Allocation concealment mechanism	18	Mechanism used to implement the random allocation sequence (eg, central computer/telephone; sequentially numbered, opaque, sealed containers), describing any steps to conceal the sequence until interventions were assigned	Pg 12
Implementation	19	Whether the personnel who enrolled and those who assigned participants to the interventions had access to the random allocation sequence	Pg12-13
Blinding	20a	Who was blinded after assignment to interventions (eg, participants, care providers, outcome assessors, data analysts)	Pg 14
	20b	If blinded, how blinding was achieved and description of the similarity of interventions	Pg 14
Statistical methods	21a	Statistical methods used to compare groups for primary and secondary outcomes, including harms	Pg 14
	21b	Definition of who is included in each analysis (eg, all randomised participants), and in which group	Pg 14
	21c	How missing data were handled in the analysis	No missing data
	21d	Methods for any additional analyses (eg, subgroup and sensitivity analyses), distinguishing prespecified from post hoc	Pg 14
**Results**			
Participant flow, including flow diagram	22a	For each group, the numbers of participants who were randomly assigned, received intended intervention, and were analysed for the primary outcome	Pg 19 (Table 1)
	22b	For each group, losses and exclusions after randomisation, together with reasons	No exlusions after randomisation
Recruitment	23a	Dates defining the periods of recruitment and follow-up for outcomes of benefits and harms	Not mentioned in the manuscript
	23b	If relevant, why the trial ended or was stopped	Not mentioned in the manuscript
Intervention and comparator delivery	24a	Intervention and comparator as they were actually administered (eg, where appropriate, who delivered the intervention/comparator, how participants adhered, whether they were delivered as intended (fidelity))	Pg 12
	24b	Concomitant care received during the trial for each group	Pg 12
Baseline data	25	A table showing baseline demographic and clinical characteristics for each group	Pg 19
Numbers analysed, outcomes and estimation	26	For each primary and secondary outcome, by group:<br>● the number of participants included in the analysis<br>● the number of participants with available data at the outcome time point<br>● result for each group, and the estimated effect size and its precision (such as 95% confidence interval)<br>● for binary outcomes, presentation of both absolute and relative effect size	Pg 14
Harms	27	All harms or unintended events in each group	No harms
Ancillary analyses	28	Any other analyses performed, including subgroup and sensitivity analyses, distinguishing pre-specified from post hoc	Pg 14
**Discussion**			
Interpretation	29	Interpretation consistent with results, balancing benefits and harms, and considering other relevant evidence	Pg 6-10
Limitations	30	Trial limitations, addressing sources of potential bias, imprecision, generalisability, and, if relevant, multiplicity of analyses	Pg 10-11

Citation: Hopewell S, Chan AW, Collins GS, Hróbjartsson A, Moher D, Schulz KF, et al. CONSORT 2025 Statement: updated guideline for reporting randomised trials. BMJ. 2025; 388:e081123. https://dx.doi.org/10.1136/bmj-2024-081123
© 2025 Hopewell et al. This is an Open Access article distributed under the terms of the Creative Commons Attribution License (https://creativecommons.org/licenses/by/4.0/), which permits unrestricted use, distribution, and reproduction in any medium, provided the original work is properly cited.

*We strongly recommend reading this statement in conjunction with the CONSORT 2025 Explanation and Elaboration and/or the CONSORT 2025 Expanded Checklist for important clarifications on all the items. We also recommend reading relevant CONSORT extensions. See www.consort-spirit.org.
